# Atypical developmental trajectories of white matter microstructure in prenatal alcohol exposure: Preliminary evidence from neurite orientation dispersion and density imaging

**DOI:** 10.3389/fnins.2023.1172010

**Published:** 2023-04-24

**Authors:** Blake A. Gimbel, Donovan J. Roediger, Abigail M. Ernst, Mary E. Anthony, Erik de Water, Madeline N. Rockhold, Bryon A. Mueller, Sarah N. Mattson, Kenneth L. Jones, Edward P. Riley, Kelvin O. Lim, CIFASD, Jeffrey R. Wozniak

**Affiliations:** ^1^Department of Psychiatry and Behavioral Sciences, University of Minnesota Twin Cities, Minneapolis, MN, United States; ^2^Great Lakes Neurobehavioral Center, Edina, MN, United States; ^3^Department of Psychology, University of Rochester, Rochester, NY, United States; ^4^Department of Psychology, San Diego State University, San Diego, CA, United States; ^5^Department of Pediatrics, University of California, San Diego, San Diego, CA, United States

**Keywords:** FASD, neurodevelopment, longitudinal, corpus callosum, NODDI

## Abstract

**Introduction:**

Fetal alcohol spectrum disorder (FASD), a life-long condition resulting from prenatal alcohol exposure (PAE), is associated with structural brain anomalies and neurobehavioral differences. Evidence from longitudinal neuroimaging suggest trajectories of white matter microstructure maturation are atypical in PAE. We aimed to further characterize longitudinal trajectories of developmental white matter microstructure change in children and adolescents with PAE compared to typically-developing Controls using diffusion-weighted Neurite Orientation Dispersion and Density Imaging (NODDI).

**Materials and methods:**

Participants: Youth with PAE (*n* = 34) and typically-developing Controls (*n* = 31) ages 8–17 years at enrollment. Participants underwent formal evaluation of growth and facial dysmorphology. Participants also completed two study visits (17 months apart on average), both of which involved cognitive testing and an MRI scan (data collected on a Siemens Prisma 3 T scanner). Age-related changes in the orientation dispersion index (ODI) and the neurite density index (NDI) were examined across five corpus callosum (CC) regions defined by tractography.

**Results:**

While linear trajectories suggested similar overall microstructural integrity in PAE and Controls, analyses of symmetrized percent change (SPC) indicated group differences in the timing and magnitude of age-related increases in ODI (indexing the bending and fanning of axons) in the central region of the CC, with PAE participants demonstrating atypically steep increases in dispersion with age compared to Controls. Participants with PAE also demonstrated greater increases in ODI in the mid posterior CC (trend-level group difference). In addition, SPC in ODI and NDI was differentially correlated with executive function performance for PAE participants and Controls, suggesting an atypical relationship between white matter microstructure maturation and cognitive function in PAE.

**Discussion:**

Preliminary findings suggest subtle atypicality in the timing and magnitude of age-related white matter microstructure maturation in PAE compared to typically-developing Controls. These findings add to the existing literature on neurodevelopmental trajectories in PAE and suggest that advanced biophysical diffusion modeling (NODDI) may be sensitive to biologically-meaningful microstructural changes in the CC that are disrupted by PAE. Findings of atypical brain maturation-behavior relationships in PAE highlight the need for further study. Further longitudinal research aimed at characterizing white matter neurodevelopmental trajectories in PAE will be important.

## 1. Introduction

Prenatal alcohol exposure (PAE) impacts the developing brain widely (Jones and Smith, 1973; Mattson et al., 2010b; Riley et al., 2011), resulting in cognitive, behavioral, and adaptive functioning difficulties. The term fetal alcohol spectrum disorder (FASD) describes a group of neurodevelopmental disorders that result from PAE. Subtypes of FASD are defined based on the presence of alcohol exposure, growth deficits, cognitive impairment, and facial dysmorphology. There are numerous diagnostic systems of FASD (Astley and Clarren, 2000; Bertrand et al., 2005; Chudley et al., 2005) including the one used here which parses FASD into fetal alcohol syndrome (FAS), partial fetal alcohol syndrome (PFAS), and alcohol-related neurodevelopmental disorder (ARND) (Hoyme et al., 2016; Wozniak et al., 2019). FASDs are highly prevalent (Lange et al., 2018; May et al., 2018) and yet often unrecognized and misdiagnosed (Chasnoff et al., 2015; McLennan, 2015).

White matter diffusion abnormalities are often seen in individuals with PAE, including lower fractional anisotropy (FA), higher mean diffusivity (MD), and higher radial diffusivity (RD) across a majority of white matter pathways, and the corpus callosum (CC) has been particularly implicated in PAE (Sherbaf et al., 2019). Several studies have also reported shape abnormalities and volume reductions in the splenium, genu, and isthmus of the CC even after controlling for total brain size (Riley et al., 1995; Lebel et al., 2008; Sherbaf et al., 2019). These abnormalities in CC volume, shape, and diffusion have been associated with poorer performance on tasks of eyeblink conditioning (Fan et al., 2015), working memory (Wozniak et al., 2009), mathematics (Lebel et al., 2010), language and reading skill (Treit et al., 2013), and interhemispheric information transfer (Biffen et al., 2022), as well as other features of FASD such as the presence of the characteristic facial features (Astley et al., 2009; Fryer et al., 2009; Li et al., 2009). Results of cross-sectional investigations suggest steeper age-related increases in FA in controls compared to participants with PAE (Treit et al., 2017) and sex-specific trajectory abnormalities potentially related to neuroendocrine factors (Uban et al., 2017). Emerging data from longitudinal studies suggest atypical brain development trajectories in PAE (Moore and Xia, 2021), highlighting the need for further study. Longitudinal findings from DTI have suggested atypical rate and timing of age-related microstructural changes across several white matter tracts in PAE (Treit et al., 2013; Kar et al., 2022). To date, studies have relied primarily on traditional diffusion tensor imaging (e.g., FA and MD) to examine white matter abnormalities in PAE. These techniques provide insight into general white matter integrity without identifying more detailed underlying mechanisms of reduced microstructural integrity (e.g., differences in density or bending and fanning of axons). In addition, DTI is unable to handle complex microstructure architectures, such as crossing fibers. Using novel diffusion modeling approaches may identify potential white matter microstructural abnormalities with greater specificity (Sherbaf et al., 2019; Figley et al., 2021).

In this study, we aimed to further explore potential atypicality in age-related corpus callosum white matter diffusion trajectories in youth with PAE using Neurite Orientation Dispersion and Density Imaging (NODDI) (Zhang et al., 2012), a diffusion MRI method that models multiple biophysically-relevant “compartments” of tissue water: intra-neurite, extra-neurite, and free water. We also aimed to explore potential sex-based differences in corpus callosum white matter diffusion trajectories given previous findings of sex-based differences in white matter diffusion trajectories in both typically-developing (Wang et al., 2012; Simmonds et al., 2014; Genc et al., 2018) youth and those with a history of PAE (Uban et al., 2017).

NODDI metrics of interest included the neurite density index (NDI), which characterizes the density of axons and dendrites, and the orientation dispersion index (ODI), which characterizes the alignment (dispersion) of tissue fibers (Mah et al., 2017; Kamiya et al., 2020). Given the close correspondence between NODDI metrics and underlying white matter integrity, this novel approach is well-suited for exploring potentially atypical white matter microstructure trajectories in youth with neurodevelopmental conditions (Young et al., 2019; Kamiya et al., 2020), such as FASD. NODDI metrics have been shown to have high scan-rescan reliability comparable to traditional diffusion metrics (Huber et al., 2019; Andica et al., 2020) and are known to correlate well with histology (Parvathaneni et al., 2018; Andica et al., 2020; Lucignani et al., 2021) – in some cases more strongly than traditional DTI metrics such as FA (Schilling et al., 2018). To our knowledge, age-related changes in white matter microstructure using NODDI have not yet been studied in PAE. We hypothesized that youth with PAE would demonstrate atypical linear trajectories of age-related change in white matter microstructure, as well as atypicality in the developmental timing and magnitude of change in microstructure, compared to unexposed, typically-developing controls (Riley et al., 1995; Lebel et al., 2008; Sherbaf et al., 2019). We also hypothesized that PAE and Control participants would show differences in correlations between age-related change in NODDI metrics and cognitive performance, consistent with prior findings (Wozniak et al., 2009; Lebel et al., 2010; Fan et al., 2015).

## 2. Materials and methods

### 2.1. Participants

All participants enrolled in this study were part of the Collaborative Initiative on Fetal Alcohol Spectrum Disorders (CIFASD) study^1^ (Mattson et al., 2010a). Participants included children with PAE (*n* = 46) and non-exposed Controls (*n* = 49). Participants were enrolled between 2017 and 2019, with the last follow-up visit occurring in 2021. Staff recruited participants with PAE from the University of Minnesota Fetal Alcohol Spectrum Disorder Clinic and other local clinics *via* brochures, letters, and self-referral. Control participants were recruited from study brochures, letters, advertisements, local community events, and mailings to Control participants from previous studies. All participants were screened by telephone. Trained staff administered IRB-approved consent and assent procedures to the participants and their guardian or parent at enrollment. Participants were compensated for study participation.

Research staff conducted phone screens and reviewed records (i.e., retrospective maternal report, social service, legal, and/or medical records) to determine PAE history of participants. Inclusion criteria for the PAE group included evidence of heavy PAE, defined as 13 or more drinks per week or 4 or more drinks per occasion at least once per week during pregnancy. Some individuals who had “suspected” but unconfirmed exposure were included if they met diagnostic criteria for pFAS or FAS based on dysmorphology and growth characteristics. Individuals who had minimal to no PAE based on available records and did not meet diagnostic criteria were excluded from the PAE group.

An expert dysmorphologist (KLJ) who had not previously met participants and who was blinded to the child’s status (PAE or Control) completed a standardized physical evaluation. Twelve participants were not able to be evaluated by KLJ due to COVID-19 pandemic restrictions. These participants were instead evaluated by the principal investigator (JRW), who has been trained by KLJ over several years and was also blinded to the child’s status. Afterward, photographs and physical measurements of each of these 12 cases were reviewed together by JRW and KLJ to reach a final consensus. For all participants, the physical assessment included the following components: ratings of the vermillion border of the upper lip and the philtrum; measurement of the palpebral fissure length (PFL), occipital-frontal circumference (OFC), height, and weight. Normative data from the University of Washington’s 4-Digit Diagnostic System was used for the vermillion and philtrum (Astley, 2004), Stromland data for PFL (Stromland et al., 1999), Nellhaus data for head circumference percentiles (Nellhaus, 1968), and the Centers for Disease Control and Prevention (CDC) growth chart data for height and weight (Kuczmarski et al., 2000). The Modified Institute of Medicine criteria were used for FASD diagnostic classification (Hoyme et al., 2016). Standardized neuropsychological assessments and parent-rated measures were administered to characterize neurobehavioral functioning, including global intellectual ability, behavioral and self-regulation abilities, other cognitive functions (memory, executive functioning, and visual spatial processing), and adaptive functioning. Consistent with Hoyme criteria (Hoyme et al., 2016), participants were considered “impaired” in a neurobehavioral domain if the standardized score was less than or equal to 1.5 standard deviations (SD) below the normative mean. Participants in the PAE group had either an intellectual impairment (IQ 1.5 standard deviations or more below the normative mean) or impairment in two or more domains of functioning (scores more than 1.5 standard deviations below the normative mean). The requirement of two impaired domains is slightly more conservative than the Hoyme criteria, which requires impairment in only one domain.

For both groups, exclusion criteria included the following: presence of a neurological or developmental disorder (e.g., epilepsy or autism spectrum disorder), severe psychiatric disabilities that would prevent participation (e.g., psychosis or mania), drug or alcohol use by the participant, very low birthweight (<1,500 grams), and contraindications to MRI scanning (e.g., non-MR-safe medical devices, braces, or claustrophobia). Prenatal exposure to substances other than alcohol (e.g., methamphetamine, marijuana, cocaine, tobacco, etc.) was not exclusionary for the PAE group because it is the most common presenting pattern. Prenatal alcohol and drug exposure (excluding tobacco and caffeine) was an exclusion criterion for the Control group.

A total of 95 participants were enrolled between the ages of 8 and 17 years at their first visit. Over the course of the longitudinal study, 25 participants failed to contribute either a baseline scan or a second scan. Loss to follow-up (which was exacerbated by the COVID-19 pandemic) was due to: inability to contact (7 PAE, 6 Control), MRI contraindications (e.g., braces; 4 PAE, 4 Control), concern about time commitment (2 Control) and no reason provided (1 PAE, 1 Control). Four participants were excluded from subsequent analysis due to image quality concerns: one due to susceptibility artifact from a dental appliance (control), one due to operator error during image acquisition (control), and two due to excessive head motion and/or aberrant FreeSurfer processing (PAE). 65 participants (PAE = 34; Control = 31) who contributed at least one scan were included in the primary analyses. Table 1 contains demographic information for the participants.

**Table 1:** Demographic characteristics of participants included in primary analyses.

	PAE (*n* = 34)	Control (*n* = 31)	Statistical Test
Age [*M* (SD)]*	12.32 (2.41)	12.45 (2.69)	*t*(60) = 0.20, *p* = 0.841
Intelligence Quotient [*M* (SD)]^†^	94.79 (15.93)	117.27 (13.90)	*t*(61) = 6.03, *p* < 0.001
Months between scans [*M* (SD)]^‡^	18.55 (3.20)	16.89 (2.38)	*t*(60) = −2.39, *p* = 0.020
Sex [*n* (%Female)]	17 (50%)	15 (48%)	𝝌^2^ = 0.02, *p* = 0.897
Ethnicity [*n* (%Hispanic)]	0	2 (6%)	𝝌^2^ = 2.26, *p* = 0.133
Race^§^			
[*n* (%American Indian/Alaska Native)]	3 (8%)	0	𝝌^2^ = 5.33, *p* = 0.021
[*n* (%Asian)]	1 (3%)	0	𝝌^2^ = 1.92, *p* = 0.166
[*n* (%Black or African American)]	11 (32%)	0	𝝌^2^ = 15.80, *p* < 0.001
[*n* (%White)]	15 (44%)	30 (97%)	𝝌^2^ = 21.12, *p* < 0.001
[*n* (%Multiracial)]	4 (12%)	1 (3%)	𝝌^2^ = 4.16, *p* = 0.041
Handedness [*n* (%Right)]‖	26 (76%)	26 (84%)	𝝌^2^ = 1.14, *p* = 0.565
Physical characteristics			
^a^Growth Deficiency	3 (9%)	2 (6%)	𝝌^2^ = 0.13, *p* = 0.720
^b^Microcephaly	3 (9%)	0	𝝌^2^ = 2.67, *p* = 0.101
^c^Dysmorphic Face	7 (21%)	2 (6%)	𝝌^2^ = 2.40, *p* = 0.122

Demographics are from the 65 participants included in the primary analyses. Age range at baseline evaluation ranged from 8 to 17 years. PAE, Prenatal Alcohol Exposure group. *Age at baseline evaluation. ^†^Intelligence Quotient as measured during the baseline evaluation. ^‡^For the calculation of group means for duration between scans in months, the mean value was substituted for cases with missing data (i.e., cases for whom only one diffusion scan was available). ^§^Chi square tests reflect comparisons of each racial group to the proportion of participants who identified as White (e.g., proportion of participants who identified as Multiracial to proportion of those who identified as White). ‖Handedness information was not available for 5 participants (4 PAE, 1 Control). ^a^Height or weight ≤ 10%ile. ^b^Head circumference ≤ 10%ile. ^c^At least two of the following: Palpebral Fissure Length ≤ 10%ile, thin vermillion border, smooth philtrum (4 or 5 on lipometer scale). The two Control participants who had “dysmorphic faces” had scores of 4 on the philtrum and 4 on the vermillion border; neither had any other facial features nor abnormal growth parameters.

Subset analyses examining symmetrized percent change (SPC) were conducted with participants for whom full MRI datasets including diffusion data from both time points were available. A total of 59 participants who completed follow-up diffusion MRI scanning (PAE = 30; Control = 29) were included in the subset analyses of SPC in NODDI diffusion metrics.

### 2.2. Evaluations

Participants were asked to complete two visits (with a target inter-scan interval of 15 months; actual interval: mean = 17.76; SD = 3.09) at the University of Minnesota. At the first visit, trained staff administered the full Wechsler Intelligence Scale for Children, 5th Edition [WISC-V; (Wechsler, 2014)]; the Trail Making, Verbal Fluency, and Color-Word Interference subtests from the Delis-Kaplan Executive Functioning System [D-KEFS; (Delis et al., 2001)]; and the Dimensional Change Card Sort and Flanker tests from NIH toolbox (Weintraub et al., 2013). At the follow-up visit, participants completed only the Digit Span, Coding, and Symbol Search subtests of the WISC-V or the Wechsler Adult Intelligence Scale 4th Edition (WAIS-IV) (Wechsler, 2008) if they were 17 years old, the Trail Making and Verbal Fluency subtests from the D-KEFS, and the NIH toolbox tasks. These cognitive assessments measure working memory, visual-motor processing speed and visual attention, number and letter sequencing, inhibitory control, and cognitive flexibility.

### 2.3. MRI acquisition and processing

Participants completed MRI scans at the University of Minnesota’s Center for Magnetic Resonance Research on a Siemens 3 T Prisma scanner (Siemens, Erlangen, Germany) equipped with a standard 32-channel head coil. The MRI acquisition matched the structural portion of the Lifespan Human Connectome Project Development (HCP-D) project (Harms et al., 2018). The T1-weighted and T2-weighted scans were acquired at each visit (baseline and follow-up) using custom pulse sequences that included automatic real-time motion detection and k-space line rejection and replacement software (Tisdall et al., 2012). The diffusion weighted scans were acquired using multiband pulse sequence, gradient tables and acquisition strategy used by HCP-D. Key scan parameters used in the MRI scans are shown in Table 2.

**Table 2:** MRI acquisition parameters.

Sequence	Imaging parameters
T1-weighted	Multi-echo MP-RAGE sequence with TR = 2,500 ms, TE = 1.8/3.6/5.4/7.2 ms, TI = 1,000 ms, voxel size = 0.8 mm isotropic, flip angle = 8 degrees
T2-weighted	SPACE sequence with TR = 3,200 ms, TE = 564 ms, voxel size = 0.8 mm isotropic, variable flip angle
Diffusion-weighted	Multi-band EPI sequence with TR = 3,230 ms, TE = 89.2 ms, voxel size = 1.5 mm isotropic, MB = 4. Four consecutive runs of about 5:31 each were acquired as 2 pairs with opposite phase encoding (A to P, P to A). Each scan had 2 shells (b = 1,500 and 3,000 s/mm^2^) and 46 directions each and 7 volumes with b = 0 s/mm^2^.

TR, repetition time; TE, echo time; MB, multiband; ms, milliseconds.

The HCP Minimal Preprocessing Pipelines (v4.0.1) were used to preprocess the imaging data (Glasser et al., 2013). For structural images, this processing included alignment between the T1-weighted and T2-weighted volumes, bias field and gradient distortion corrections, and registration of the data to MNI space followed by volume segmentation and surface parcellation using FreeSurfer (v6.0.0). Subsequently, we utilized FreeSurfer’s longitudinal processing stream wherein an unbiased within-subject template space and image are created (Reuter et al., 2010; Reuter and Fischl, 2011) and several steps of the FreeSurfer processing are reinitialized with common information from the within-subject template. This approach results in improved reliability over standard cross-sectional processing (Reuter et al., 2012). For diffusion-weighted images, HCP preprocessing included rigid AC-PC alignment to the structural images, correction for susceptibility related distortions using FSL’s “topup” (Andersson et al., 2003; Smith et al., 2004), and eddy current and movement-induced distortion correction using FSL’s “eddy” tool (v6.0.1) (Andersson and Sotiropoulos, 2016). For improved performance, eddy was run with the optional slice outlier replacement, slice-to-volume motion correction and susceptibility-by motion correction routines (Andersson et al., 2016, 2017, 2018).

**Figure 1:**
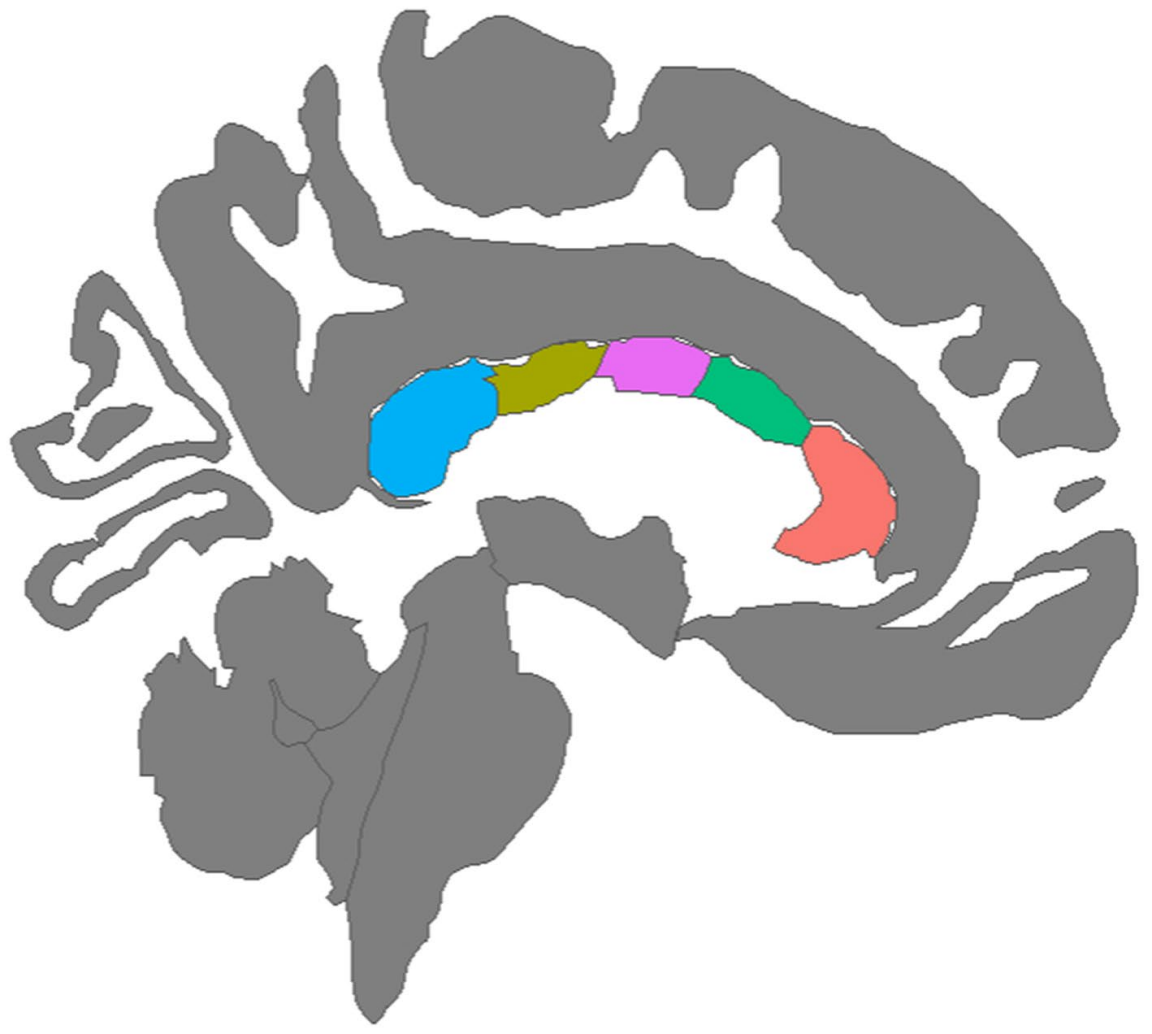
Mid-sagittal view of five corpus callosum tracts generated by tractography: (left to right) Blue = Posterior, Olive = Mid Posterior, Purple = Central, Green = Mid Anterior, Orange = Anterior.

To assess white matter microstructure, the Neurite Orientation Dispersion and Density Imaging model (NODDI) (Zhang et al., 2012) was applied to the diffusion signal. Voxel-wise NODDI metrics were estimated using the Accelerated Microstructure Imaging *via* Convex Optimization (AMICO) toolkit (Daducci et al., 2015). Mean NODDI parameters were calculated within each of the five corpus callosum segments identified by the FreeSurfer longitudinal stream by registering the white matter segmentations to the AC-PC aligned diffusion images then extracting regional averages for each scalar metric using FreeSurfer’s mri_segstats function. A graphical depiction of the five CC regions was created with the ggseg package in the R library (Figure 1; Mowinckel and Vidal-Piñeiro, 2020). An experienced rater (DJR) visually inspected all structural images and NODDI scalar maps, checked for aberrant FreeSurfer processing using ENIGMA quality control protocols (Thompson et al., 2020), and ensured that movement-induced artifacts in diffusion images were adequately resolved during preprocessing.

Longitudinal changes in both the orientation dispersion index (ODI) and the neurite density index (NDI) were evaluated. NDI models the intracellular fraction of the tissue, reflecting the density of white matter axonal fibers and possibly dendritic projections (Zhang et al., 2012). Well-established relationships between dendritic arbor complexity and cognitive function suggest that neurite density is functionally relevant (Kaufmann and Moser, 2000). Higher NDI values in white matter are thought to reflect axonal growth, greater axonal density, and/or myelination (Mah et al., 2017). In contrast, ODI models the extracellular fraction of tissue (between axons) and, thus, reflects the degree of bending and fanning of axons in white matter as represented by the angular variation of diffusion orientation (Zhang et al., 2012).

### 2.4. Statistical analyses

Using the “stats” package in RStudio version 4.1.1 (R Core Team, 2021), chi-square and independent samples *t*-tests were used to test group differences in demographic characteristics. Longitudinal changes in NODDI diffusion metrics (i.e., ODI and NDI) for each of the five corpus callosum regions were evaluated with two separate approaches. First, linear mixed effects models were evaluated using the “lme4” (Bates et al., 2015) and “lmerTest” (Kuznetsova et al., 2017) packages in RStudio. As linear mixed effects models are robust to missing data, we used this approach so that participants with missing diffusion data at one time point could be included (thus boosting statistical power). Linear (y = age + sex + age × diagnostic group + sex × diagnostic group [1 | subject]) terms were modeled. Age, sex, diagnostic group, and interactions were modeled as fixed factors with random subject-specific intercepts. Restricted maximum likelihood (REML) estimation was set to true. Interaction terms that were not significant or at the trend-level were removed and the model was run without them. False discovery rate (FDR) was used to correct for 10 multiple comparisons for analyses of NDI and ODI (5 CC regions and 2 diffusion metrics). FDR was also used to correct for 10 multiple comparisons for analyses of NDI and ODI using general linear models as described below (5 CC regions and 2 diffusion metrics). FDR correction was performed for the entire family of analyses together (i.e., linear mixed effects models and general linear models). Significance for these analyses was set at *q* < 0.05. *Post hoc* analyses (i.e., to further examine main effects for group and sex) were performed using analyses of covariance (with inter-scan interval as a covariate) and Tukey’s Test for multiple comparisons with the “stats” package. Importantly, although white matter microstructure maturation is known to be non-linear across larger developmental timescales (Lebel et al., 2019), given the relatively narrow duration between scans (17 months on average) in this study, the modest sample size (PAE = 34; Control = 31), and the novel, exploratory nature of our investigation of NODDI with this population, linear models of microstructure diffusion trajectories were believed to be appropriate.

In alternate analyses of a subset of participants with two scans, we examined symmetrized percent change (SPC), a dimensionless measure of change (Berry and Ayers, 2006; Reuter et al., 2012) across two time points for the 59 participants for whom both baseline and follow-up diffusion MRI scans were available (PAE = 30; Control = 29). SPC was calculated as:


$$SPC=\frac{\left(Scan2-Scan1\right)}{\left(Scan2+Scan1\right)}\times 100$$

where Scan1 represents diffusion values at baseline scan, and Scan2 represents diffusion values at follow-up scan. This approach was used in order to further characterize potential group differences in the rate and timing of age-related changes in white matter microstructure. For these analyses, we used the “stats” package in RStudio version 4.1.1 (R Core Team, 2021). SPC values across five regions of the corpus callosum were computed for each participant from the two time points. General linear models were performed with SPC in ODI/NDI as the dependent variable and age, sex, diagnostic group (PAE vs. Control), age-by-group, and sex-by-group interactions as predictors. Interaction terms that were not significant or at the trend-level were removed and the model was run without them. Mean age (i.e., the midpoint between the two scans) was used in general linear models to be consistent with the fact that SPC represents two time points. Although mean age was investigated as both a linear and quadratic (i.e., age ^2) variable in SPC analyses, no meaningful difference between variables with linear versus quadratic age was observed. Therefore, the more parsimonious models using linear age were used.

Exploratory analyses were performed with Pearson correlations to investigate the relationship between SPC in corpus callosum diffusion metrics across all five regions and executive function test performance at follow-up testing. Separate analyses were completed independently for each diagnostic group (PAE vs. Control). False discovery rate (FDR) was used to correct for 150 multiple comparisons for PAE participants (15 executive function tasks and SPC in two diffusion metrics [NDI and ODI] across five CC regions), and 150 multiple comparisons for Control participants (15 executive function tasks and SPC in two diffusion metrics [NDI and ODI] across five CC regions). Significance for these analyses was set at *q* < 0.05. Correlations between all executive function measures and SPC across the five regions of the CC were visualized with the “corrplot” package (Wei and Simko, 2017).

Lastly, exploratory analyses were performed with independent sample t-tests to examine the relationship of symmetrized percent change in corpus callosum microstructure to facial dysmorphology across the entire sample. Specifically, facial features (i.e., palpebral fissure length, upper lip vermillion border, and philtrum) rated at the baseline evaluation were categorized into dichotomous variables (i.e., dysmorphic vs. non-dysmorphic). Comparisons were examined between participants with and without dysmorphic faces (i.e., two of the following: palpebral fissure length ≤ 10%ile; scores of 4 or 5 on ratings of the upper lip vermillion border and philtrum), as well as comparisons between participants with and without dysmorphology in each facial feature (i.e., palpebral fissure length, upper lip, philtrum). Given the exploratory nature of these analyses, results were not corrected for multiple comparisons.

## 3. Results

### 3.1. Participant characteristics

As shown in Table 1, the PAE and Control groups did not differ significantly with respect to demographic variables including age, sex, ethnicity, and handedness, although there were differences in race with more individuals identifying as non-white in the PAE group. As expected, the PAE group had lower mean IQ scores (*M* = 94.79; average range) compared to the Control group (*M* = 117.27; high average range–a mean score difference of approximately 22 points). Estimates of head motion during diffusion scans (FSL Eddy-derived metric) were not significantly different between diagnostic groups at baseline (*t*[61] = 0.96, *p* = 0.339) or follow-up scan (*t*[45] = −0.82, *p* = 0.418). Despite efforts to complete follow-up MRIs within 15-months of the baseline scan for all participants, the PAE group demonstrated a slightly longer mean inter-scan interval than Controls (approximately two months longer on average). There were no outliers in terms of inter-scan interval. Inter-scan interval (months) was not significantly correlated with symmetrized percent change in NDI in any CC region and was only correlated significantly with ODI in one of five CC regions (mid posterior) (r [57] = 0.28, *p* = 0.029). Duration between scans was not significantly correlated with executive function performance at follow-up testing. Therefore, we did not consider the inter-scan interval to be a confounding variable. However, for significant or trend-level findings in SPC in ODI in the mid posterior region of the CC, we conservatively performed additional analyses with the inclusion of inter-scan interval as a covariate. We also conservatively included the inter-scan interval as a covariate in *post hoc* analyses to further characterize the group and sex differences in absolute NDI and ODI values at baseline and follow-up scan.

### 3.2. Neurite density index

**Figure 2:**
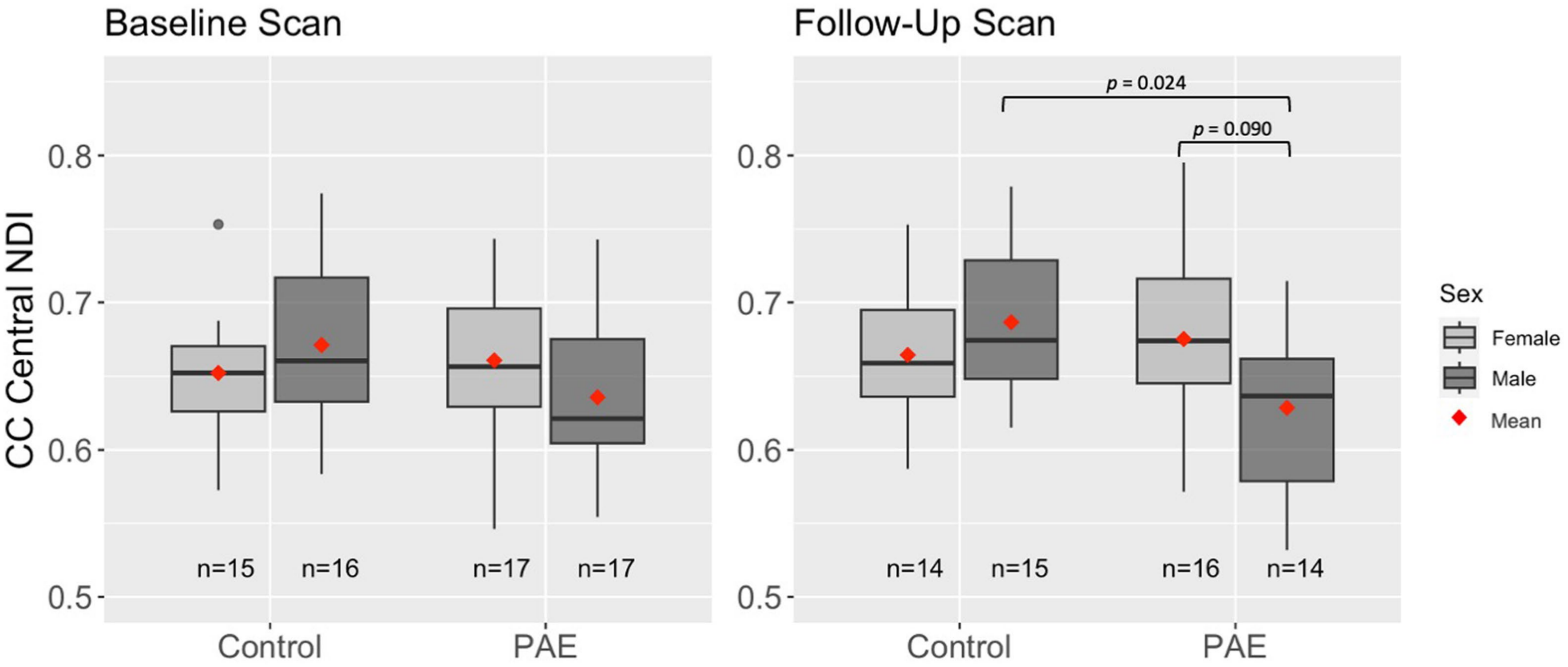
Differences in mean NDI in the central CC at baseline and follow-up scan by group and sex. Results are displayed for linear mixed effects models examining group differences (PAE vs. Control) in age-related trajectories of corpus callosum white matter microstructure. Interactions that were not significant or trend-level were removed from the model.

A main effect for age was observed for NDI across all five CC regions, where NDI increased linearly with age across diagnostic groups (Table 3). There were no significant main effects for group (PAE vs. Control) or sex (male vs. female). However, there was a trend-level sex-by-group interaction (*p* = 0.078) for NDI in the central CC. *Post hoc* analyses revealed that male participants in the PAE group (*M* = 0.64) demonstrated marginally lower mean NDI in the central CC at baseline scan compared to female PAE participants (*M* = 0.66) and both male (*M* = 0.67) and female (*M* = 0.65) Control participants. At follow-up scan, male PAE participants (*M* = 0.63) had lower mean NDI in the central CC compared to female PAE participants (*M* = 0.68), measured at the trend level (*p* = 0.090), and significantly lower mean NDI compared to male Controls (*M* = 0.69; *p* = 0.024) (Figure 2). No significant age-by-group interactions were observed for NDI across CC regions.

**Table 3:** Linear mixed effects model results for NDI.

Neurite Density Index (NDI)	PE (SE)	*t*	*p*
Anterior CC			
Intercept	0.67 (0.02)	28.49	<0.001
Age	0.00 (0.00)	2.66	0.009*
Sex	0.01 (0.01)	1.12	0.265
Group	−0.00 (0.01)	−0.88	0.383
Mid Anterior CC			
Intercept	0.59 (0.02)	24.49	<0.001
Age	0.01 (0.00)	3.31	0.001*
Sex	−0.00 (0.01)	−0.16	0.871
Group	−0.00 (0.01)	−0.13	0.899
Central CC			
Intercept	0.56 (0.02)	25.45	<0.001
Age	0.01 (0.00)	4.48	<0.001*
Sex	0.00 (0.01)	0.49	0.629
Group	0.01 (0.01)	1.40	0.165
Sex-by-group	−0.01 (0.01)	−1.79	0.078
Mid Posterior CC			
Intercept	0.59 (0.02)	31.27	<0.001
Age	0.01 (0.00)	3.95	<0.001*
Sex	0.00 (0.00)	0.12	0.907
Group	0.00 (0.00)	0.98	0.330
Posterior CC			
Intercept	0.70 (0.02)	35.44	<0.001
Age	0.01 (0.00)	3.49	<0.001*
Sex	0.00 (0.00)	0.42	0.677
Group	−0.00 (0.01)	−0.03	0.979

Results are displayed for linear mixed effects models examining group differences (PAE vs. Control) in age-related linear trajectories of corpus callosum white matter microstructure. Significant main effects for age and sex that survived FDR correction are indicated with bold text and with *. Interactions that were not significant or trend-level were removed from the model. Abbreviations: PE, parameter estimate; SE, standard error; CC, corpus callosum.

### 3.3. Orientation dispersion index

A main effect for age was observed for ODI across the central, mid posterior, and posterior CC regions, where ODI increased linearly with age across diagnostic groups (Table 4). Similarly, a trend-level main effect for age was observed in the anterior CC. There was not a significant effect for group (PAE vs. Control) across any CC regions. However, there was a main effect for sex (*p* = 0.001; *q* = 0.004) in the central CC and the mid posterior CC (*p* = 0.044; *q* = 0.117). *Post hoc* analyses revealed that, collapsing across PAE and Control groups, male participants demonstrated significantly higher mean ODI in the central CC at baseline scan (*M* = 0.049) compared to female participants (*M* = 0.047), *F*(1, 62) = 7.49, *p* = 0.008. Similarly, at the follow-up scan, male participants (*M* = 0.050) had significantly higher mean central CC ODI compared to female participants (*M* = 0.047), *F*(1, 56) = 9.18, *p* = 0.004. Of note, group mean differences are here reported at the third decimal place for clarity. No significant age-by-group or sex-by-group interactions were observed for ODI across CC regions.

**Table 4:** Linear mixed effects model results for ODI.

Orientation Dispersion Index (ODI)	PE (SE)	*t*	*p*
Anterior CC			
Intercept	0.04 (0.00)	27.94	<0.001
Age	0.00 (0.00)	1.79	0.077
Sex	−0.00 (0.00)	−0.60	0.553
Group	−0.00 (0.00)	−0.07	0.941
Mid Anterior CC			
Intercept	0.05 (0.00)	23.67	<0.001
Age	0.00 (0.00)	1.44	0.151
Sex	−0.00 (0.00)	−1.11	0.269
Group	−0.00 (0.00)	−0.40	0.694
Central CC			
Intercept	0.04 (0.00)	26.70	<0.001
Age	0.00 (0.00)	2.74	0.007*
Sex	−0.00 (0.00)	−3.32	0.001*
Group	−0.00 (0.00)	−0.31	0.755
Mid Posterior CC			
Intercept	0.04 (0.00)	37.53	<0.001
Age	0.00 (0.00)	3.00	0.003*
Sex	−0.00 (0.00)	−2.06	*0.044*
Group	0.00 (0.00)	0.96	0.343
Posterior CC			
Intercept	0.04 (0.00)	28.32	<0.001
Age	0.00 (0.00)	3.44	<0.001*
Sex	0.00 (0.00)	0.98	0.333
Group	−0.0 (0.00)	−0.01	0.992

Results are displayed for linear mixed effects models examining group differences (PAE vs. Control) in age-related linear trajectories of corpus callosum white matter microstructure. Significant main effects for age and sex that survived FDR correction are indicated with bold text and with *. Significant main effects that did not survive FDR correction are marked in italics. Interactions that were not significant or trend-level were removed from the model. PE, parameter estimate; SE, standard error; CC, corpus callosum.

### 3.4. Symmetrized percent change in NDI

No significant main effect for age, group (PAE vs. Control), or interaction effects (mean age-by-group and sex-by-group) were found for SPC in NDI across the 5 regions of the corpus callosum (Table 5). However, a significant main effect for sex (*p* = 0.014; *q* = 0.042) was observed in the anterior CC, such that females demonstrated greater percent increases in NDI compared to males when collapsing across diagnostic groups (PAE vs. Control).

**Table 5:** General linear model results for SPC in NDI.

Neurite Density Index (NDI)	PE (SE)	*t*	*p*
Anterior CC			
Intercept	2.01 (1.74)	1.16	0.252
Age	−0.04 (0.12)	−0.34	0.733
Sex	−1.62 (0.64)	−2.55	0.014*
Group	−0.29 (0.64)	−0.46	0.646
Mid Anterior CC			
Intercept	0.37 (1.59)	0.23	0.816
Age	0.04 (0.11)	0.38	0.704
Sex	−0.67 (0.58)	−1.15	0.257
Group	0.29 (0.58)	0.50	0.618
Central CC			
Intercept	1.79 (1.61)	1.11	0.270
Age	−0.04 (0.11)	−0.32	0.748
Sex	−0.59 (0.59)	−1.00	0.321
Group	−0.46 (0.59)	−0.78	0.442
Mid Posterior CC			
Intercept	2.81 (1.78)	1.58	0.121
Age	−0.17 (0.13)	−1.32	0.193
Sex	0.00 (0.66)	0.00	0.999
Group	−0.47 (0.66)	−0.72	0.473
Posterior CC			
Intercept	−0.25 (1.22)	−0.20	0.841
Age	0.04 (0.09)	0.49	0.624
Sex	−0.19 (0.45)	−0.44	0.666
Group	0.27 (0.45)	0.61	0.545

Results are displayed for general linear models examining group differences (PAE vs. Control) in symmetrized percent change of corpus callosum white matter microstructure. Significant main effects for sex that survived FDR correction are indicated with bold text and with *. Interactions that were not significant or trend-level were removed from the model. PE, parameter estimate; SE, standard error; CC, corpus callosum.

### 3.5. Symmetrized percent change in ODI

**Figure 3:**
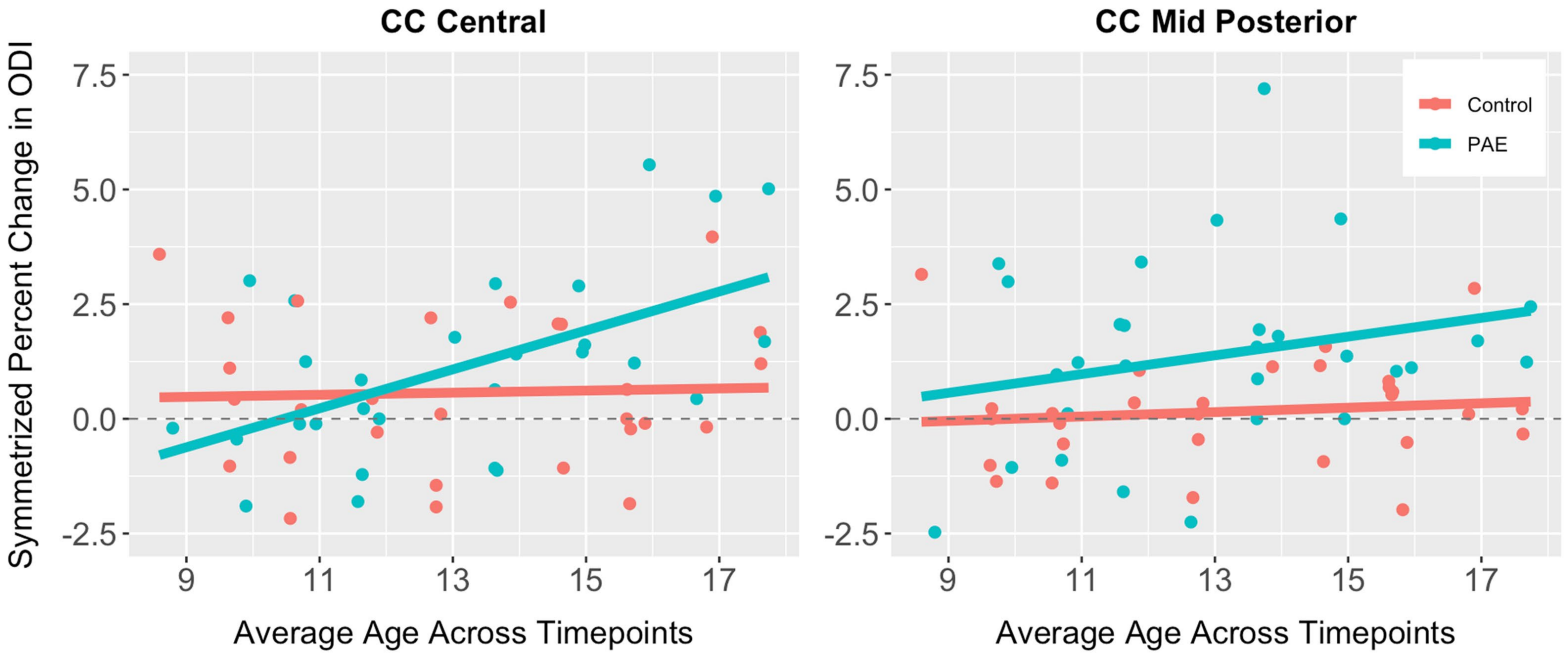
Scatterplots illustrating group differences in symmetrized percent change in ODI in the central and mid posterior corpus callosum. Results are displayed for general linear models examining group differences (PAE vs. Control) in developmental timing and degree of change in corpus callosum white matter microstructure.

No significant main effects for age or group were observed for SPC across the five CC regions (Table 6). A significant main effect of sex was observed for SPC in the anterior CC, such that females demonstrated greater percent increases in ODI compared to males when collapsing across diagnostic groups (PAE vs. Control). For four out of five regions of the CC, no interaction effects (mean age-by-group and sex-by-group) were found for SPC in ODI. However, for the fifth region (central CC), a significant mean age-by-group interaction effect was observed (Figure 3), such that PAE participants demonstrated minimal changes in ODI at younger ages, and increases in ODI at older ages compared to Controls, who showed minimal changes in ODI with age (*p* = 0.023; *q* = 0.064). In addition, a trend-level sex-by-group interaction was observed for ODI in the central CC (*p* = 0.062). That is, female participants in the Control group demonstrated reductions in central CC ODI, while male Control participants and both female and male PAE participants demonstrated increases in ODI. Lastly, a main effect for group was observed for SPC in ODI in the mid posterior CC, with PAE participants demonstrating significantly greater percent increases in ODI than Controls (*p* = 0.021; *q* = 0.061) (Figure 3). This uncorrected finding was reduced to the trend-level (*p* = 0.072) with the inclusion of inter-scan interval as a covariate.

**Table 6:** General linear model results for SPC in ODI.

Orientation Dispersion Index (ODI)	PE (SE)	*t*	*p*
Anterior CC			
Intercept	−1.81 (1.41)	−0.84	0.408
Age	0.15 (0.10)	1.48	0.144
Sex	−1.51 (0.52)	−2.90	0.005*
Group	0.24 (0.52)	0.46	0.647
Mid Anterior CC			
Intercept	−1.48 (2.03)	−0.73	0.470
Age	0.17 (0.14)	1.17	0.249
Sex	−1.00 (0.75)	−1.33	0.188
Group	0.23 (0.75)	0.31	0.759
Central CC			
Intercept	0.67 (2.06)	0.32	0.747
Age	−0.08 (0.15)	−0.53	0.601
Sex	1.50 (0.83)	1.81	0.076
Group	−5.05 (3.09)	−1.64	0.108
Age-by-Group	0.53 (0.23)	2.34	*0.023*
Sex-by-Group	−2.21 (1.16)	−1.90	0.062
Mid Posterior CC			
Intercept	−1.01 (1.26)	−0.80	0.427
Age	0.07 (0.09)	0.79	0.431
Sex	0.43 (0.46)	0.93	0.357
Group	1.10 (0.46)	2.38	*0.021*
Posterior CC			
Intercept	−0.00 (1.31)	−0.00	0.997
Age	0.04 (0.09)	0.46	0.649
Sex	−0.04 (0.48)	−0.09	0.929
Group	0.59 (0.48)	1.23	0.225

Results are displayed for general linear models examining group differences (PAE vs. Control) in symmetrized percent change of corpus callosum white matter microstructure. Significant main effects for sex that survived FDR correction are indicated with bold text and with *. Significant main effects for group that did not survive multiple comparison correction are indicated with italics. Interactions that were not significant or trend-level were removed from the model. PE, parameter estimate; SE, standard error; CC, corpus callosum.

### 3.6. Exploratory correlations between change in white matter microstructure and executive function performance

**Figure 4:**
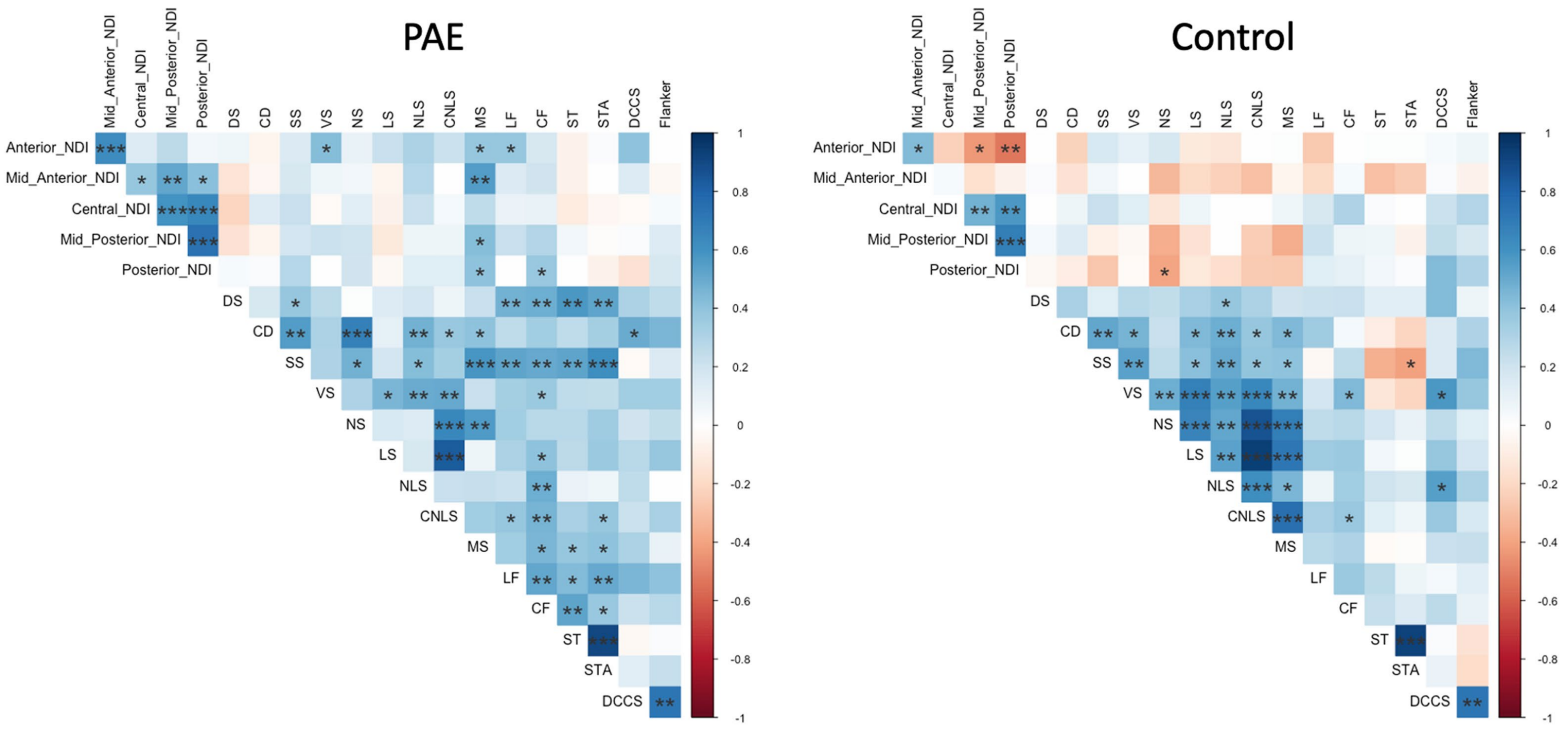
Correlation between SPC in corpus callosum NDI and executive function performance. DS, Digit Span; CD, Coding; SS, Symbol Search; *VS*, Visual Scanning; NS, Number Sequencing; LS, Letter Sequencing; NLS, Number-Letter Sequencing; CNLS, Combined Number-Letter Sequencing; MS, Motor Speed; LF, Letter Fluency; CF, Category Fluency; ST, Switching Total; STA, Switching Total Accuracy; DCCS, Dimensional Change Card Sort; blue shaded cells indicate positive correlations; red shaded cells indicate negative correlations. *p < 0.05, **p < 0.01, ***p < 0.001.

**Figure 5:**
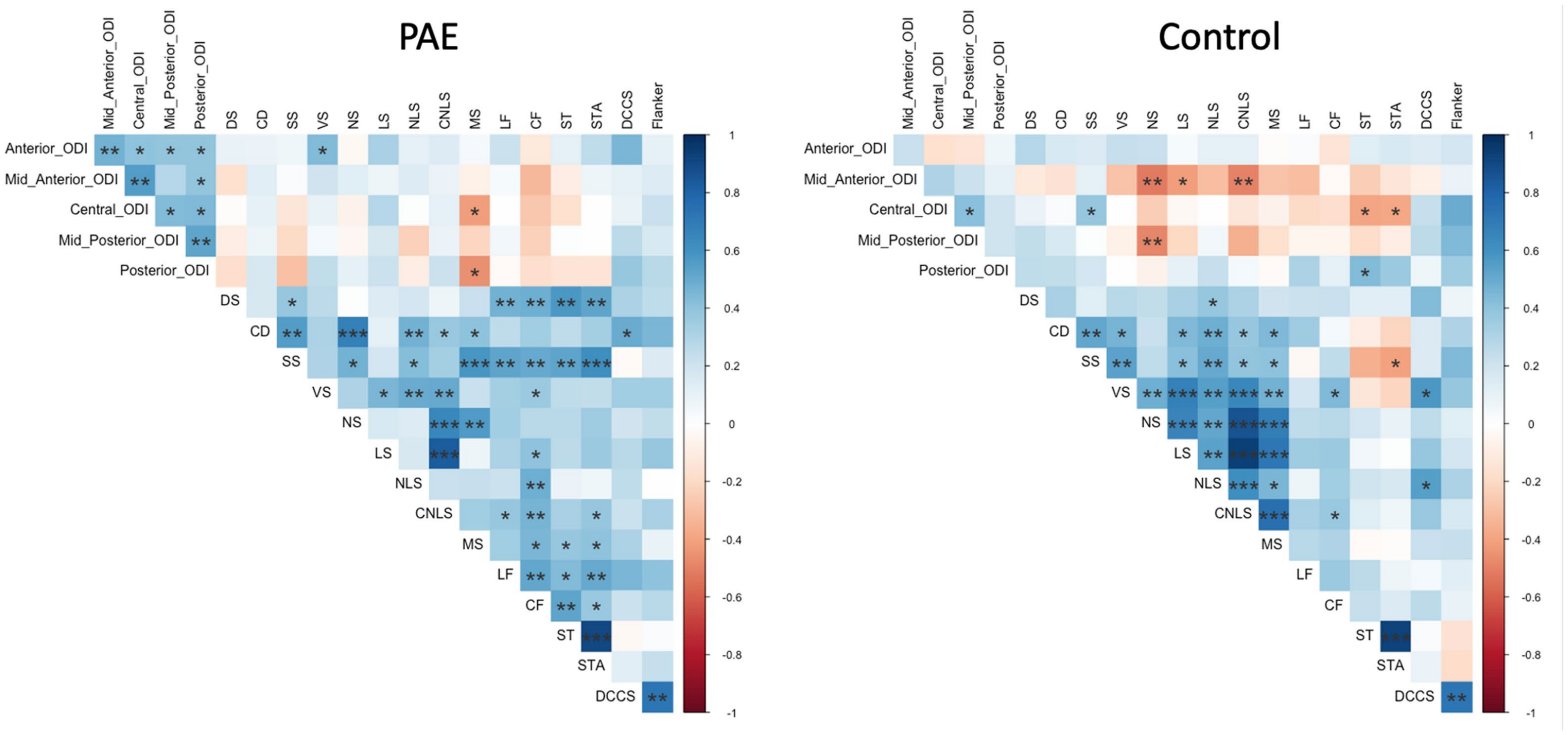
Correlation between SPC in corpus callosum ODI and executive function performance. DS, Digit Span; CD, Coding; SS, Symbol Search; *VS*, Visual Scanning; NS, Number Sequencing; LS, Letter Sequencing; NLS, Number-Letter Sequencing; CNLS, Combined Number-Letter Sequencing; MS, Motor Speed; LF, Letter Fluency; *CF*, Category Fluency; ST, Switching Total; STA, Switching Total Accuracy; DCCS, Dimensional Change Card Sort; blue shaded cells indicate positive correlations; red shaded cells indicate negative correlations. *p < 0.05, **p < 0.01, ***p < 0.001.

Exploratory analyses performed separately for each group (PAE and Control) revealed divergent patterns of associations between change in corpus callosum microstructure and executive function performance at follow-up evaluation. Correlation matrices generated separately for each group (PAE and Control) suggested Controls demonstrated a greater number of negative correlations between percent change in NDI and executive function performance compared to PAE participants, indicating that greater negative percent change in NDI (i.e., reductions in the density of white matter fibers) was associated with better performance (Figure 4). In contrast, PAE participants demonstrated a number of significant positive correlations between percent change in NDI and executive function performance. Similarly, results suggest a greater number of negative correlations between percent change in ODI and executive function performance for Controls compared to PAE participants. That is, greater negative percent change in ODI (i.e., reductions in the bending and fanning of axons) was associated with better task performance (Figure 5). No significant correlations survived FDR correction for multiple comparisons.

### 3.7. Exploratory analyses examining the relationship between white matter microstructure and facial dysmorphology

Results of independent samples *t*-tests indicated that, collapsing across diagnostic groups, there were no differences between dysmorphology groups (i.e., two of the following: palpebral fissure length ≤ 10%ile, thin upper lip vermillion border, smooth philtrum) in SPC in ODI and NDI across the five regions of the CC. However, participants with abnormal philtrum measurements (i.e., a score of 4 or 5) had greater SPC increases in ODI in the posterior CC than those with a normal philtrum, measured at the trend level, *t*(14) = −1.80, *p* = 0.094. Participants with abnormal upper lip measurements (i.e., a score of 4 or 5) had significantly greater SPC increases in ODI in the posterior CC than those with a normal upper lip, *t*(15) = −3.29, *p* = 0.005.

## 4. Discussion

The data described here show significant age effects for NDI and ODI, demonstrating that these NODDI metrics are sensitive to typical developmental changes in the central white matter during childhood and adolescence. Age was positively associated with diffusion metrics that reflect increased axonal density (NDI) and axonal organization (ODI). These data are consistent with findings of relatively steep increases in NDI with age from early childhood to adulthood and more modest (but significant) age-related increases in ODI when examining callosal white matter as well as association fibers and projection fibers (Chang et al., 2015). The authors of this study interpreted their findings in the context of prior diffusion tensor imaging studies suggesting that increases in axonal diameter and myelination may be the prominent underlying developmental processes responsible for these observed patterns. A similar study found an exponential increase in NDI during infancy and childhood but a plateauing in NDI during adolescence (Lynch et al., 2020), and relatively less change in ODI over the same developmental time frame. Consistent with these findings, Mah et al. (2017) demonstrated increases in NDI with age across the brain in typically developing children and adolescents (ages 8–13 years). However, they found no age-related increases in ODI, which they interpreted as suggesting that age-related changes in white matter microstructure are primarily driven by changes in axon density and myelination, rather than increasing axon coherence/organization. Lastly, in a large study of white matter change across the lifespan, Slater et al. (2019) also documented rapid changes in NODDI metrics during childhood and adolescence. Together, the literature and our findings demonstrate that NODDI is sensitive to typical maturational change in white matter microstructure in children and adolescents and may, therefore, be sensitive to altered developmental trajectories like those seen following PAE in other brain metrics such as cortical thickness, gyrification, and gray and white matter volumes (Moore and Xia, 2021).

Thus far, although NODDI has proven to be sensitive to frank neuropathological processes especially in white matter, such as demyelination in multiple sclerosis (Collorone et al., 2020), Wallerian degeneration in stroke (Mastropietro et al., 2019), and corticospinal lesions underlying cerebral palsy (Nemanich et al., 2019), it has not yet been applied widely to neurodevelopmental disorders (Kamiya et al., 2020). Some of the few NODDI studies to examine neurodevelopment show that preterm infants have lower cortical NDI compared to term infants (Batalle et al., 2017; Dimitrova et al., 2021) and higher ODI in white matter (suggesting organizational anomalies) compared to term infants (Blesa et al., 2020). In children born prematurely, lower NDI appears to be associated with lower IQ (Kelly et al., 2016). White matter alterations in neurofibromatosis type 1 have been also detected using NODDI (Billiet et al., 2014), and the diffusion abnormalities have been attributed to intra-myelin edema. Similarly, neonates with congenital heart disease demonstrate higher ODI in the corpus callosum and lower NDI in the corpus callosum, uncinate fasciculus, and superior fronto-occipital fasciculus compared to typically developing controls (Karmacharya et al., 2018). Another study demonstrated associations between maternal depression/anxiety and NODDI metrics in infants during the first month of life (Dean et al., 2018). Together, these studies suggest that, in addition to characterizing frank neuropathology, NODDI metrics are also sensitive to more subtle types of white matter disturbances in a range of neurodevelopmental conditions.

In the current study, we did not find overall differences in white matter microstructural integrity of the corpus callosum for participants with PAE compared to controls, but we did observe marginally lower NDI for PAE males in one region (central CC) at baseline and a significant abnormality in NDI for PAE males in the same region at follow-up. We expected to see larger, more widespread effects, given that a large number of cross-sectional studies have shown that children and adolescents with PAE display atypical white matter microstructure, particularly in the CC (Riley et al., 1995; Lebel et al., 2008; Sherbaf et al., 2019). Using DTI, we have previously shown white matter alterations in PAE in the central and posterior CC (Wozniak et al., 2006, 2009) and demonstrated associations with disrupted functional connectivity between cortical regions served by those CC regions (Wozniak et al., 2011). Importantly, to date, studies of white matter microstructure in PAE have utilized traditional diffusion metrics such as FA and MD. While these findings have shed light on important white matter anomalies associated with PAE, interpretation of metrics such as FA and MD is impacted by several limitations, including the non-specificity of water diffusion restriction, as diffusion of water in white matter fibers may be restricted by fiber diameter and density, membrane permeability, myelination, and directional organization (Figley et al., 2021). Similarly, an estimated 60 to 90 percent of white matter voxels in a given brain contain multiple fiber bundles with different orientations (Jeurissen et al., 2013). Together, these factors limit the specificity with which biologically-meaningful interpretations of traditional diffusion metrics (e.g., FA) can be made, as developmental anomalies in these metrics may be due to a wide array of biological mechanisms. The use of NODDI in this study represents a novel application of this diffusion modeling approach to investigate white matter microstructure development in PAE. As NODDI has been shown to demonstrate a closer relationship with histology (Grussu et al., 2017; Schilling et al., 2018), this approach may be particularly well-suited to characterizing atypical WM microstructure trajectories in youth with neurodevelopmental conditions such as FASD (Young et al., 2019; Kamiya et al., 2020). Findings presented here suggest that NODDI modeling may provide additional insights into atypical white matter microstructural development in PAE. Specifically, findings of the current study suggest that corpus callosum microstructural anomalies in PAE documented in previous studies may be driven primarily by disruptions in axonal coherence/organization and geometry rather than myelination and density. An important consideration for interpretation of our results include recent findings of variation of axonal tortuosity and diameter along the longitudinal axis (de Kouchkovsky et al., 2016; Abdollahzadeh et al., 2019), which may reflect age-related microstructural changes (e.g., myelination). These factors may partially explain our observations of age-related changes in ODI in this study. Future research will benefit from further characterizing the nature and developmental timing of corpus callosum microstructural anomalies associated with PAE.

While data presented here suggest linear trajectories in corpus callosum neurite density and organization are similar in participants with PAE compared to Controls, we did find that the amount and timing of changes in fiber organization differed significantly by group for the central region of the CC, and this group difference appeared to widen during adolescence. Participants with PAE demonstrated minimal changes in ODI (indexing the bending and fanning of axons) at younger ages and an atypical degree of increased ODI at older ages. In contrast, Controls demonstrated minimal changes in ODI with age. This atypically steep change in bending and fanning of axons in corpus callosum fibers during adolescence in the PAE group may suggest a “catch-up” period to compensate for delayed maturation earlier in childhood, consistent with findings from previous studies. Treit et al. (2013) evaluated DTI in a small sample of children ages 5 to 15 years who completed two or three MRI scans across approximately two to four years. Results indicated age-by-group interactions for several white matter tracts (i.e., superior longitudinal fasciculus, superior and inferior fronto-occipital fasciculus), suggesting greater reductions in mean diffusivity in the FASD group and a delayed pattern of microstructural maturation compared to unexposed controls. Using a similar longitudinal methodology, Kar et al. (2021) compared white matter microstructural trajectories in a younger sample of children with PAE and controls ages 2 to 8 years. Results indicated a flatter trajectory of change (decreases) in mean diffusivity across time in the PAE group compared to controls. Specific tracts in which age-by-group differences were observed included the genu of the corpus callosum, uncinate fasciculus, inferior longitudinal fasciculus, and inferior fronto-occipital fasciculus. The authors speculated that this delay in white matter development associated with PAE could reflect reduced brain plasticity, potentially explaining discrepancies between cross-sectional findings of lower MD in young children (e.g., Kar et al., 2021) and higher MD in older children and adolescents (e.g., Fan et al., 2016). Together, data presented here are broadly consistent with these two longitudinal studies (the only published longitudinal studies of white matter microstructure maturation in PAE conducted to date) of atypical developmental timing of white matter microstructural maturation associated with PAE. Future studies will benefit from larger sample sizes (thus increasing statistical power to detect developmental differences) and the inclusion of multiple diffusion modeling approaches in order to better characterize white matter microstructural maturation across development in PAE.

In the current study, we observed several notable differences in white matter microstructure maturation by sex, as well as several sex-by-group interactions. Specifically, linear trajectories in orientation dispersion in the central CC varied by sex at the whole-group level, suggesting that males in both groups demonstrated higher mean ODI. Analyses of symmetrized percent change also revealed sex differences in the magnitude of change in neurite density and orientation dispersion in the anterior CC, with females showing greater increases than males. These findings align with previous work suggesting sex differences in white matter development in typically-developing individuals, including relative delays in microstructural development (e.g., as measured by DTI) in males compared to females (Wang et al., 2012; Simmonds et al., 2014; Genc et al., 2018), which may reflect developmental differences by sex in the timing of puberty onset (also delayed in males) and the role gonadal hormones in white matter development (Sisk and Foster, 2004; Chavarria et al., 2014; Ho et al., 2020). In contrast, two studies examining white matter microstructure development in typically-developing samples using NODDI found no sex differences in ODI and NDI (Mah et al., 2017; Lynch et al., 2020), and a third study did not examine sex differences in maturational trajectories (Chang et al., 2015). As such, further research exploring potential sex differences in NODDI metrics is needed.

In addition to effects for sex at the whole-group level, we observed a trend-level sex-by-group interaction for linear trajectories of neurite density in the central CC. Further analyses showed that males with PAE demonstrated lower mean neurite density at both scans compared to females with PAE and both male and female Controls. A similar sex-by-group interaction was observed for symmetrized percent change in ODI in the central CC, with female Control participants showing greater reductions in ODI in the central CC compared to male Controls and both female and male PAE participants. A range of sex differences have been documented in animal models of PAE including differences in behavioral functioning and in hypothalamic–pituitary–adrenal axis and neurotransmitter function (Sliwowska et al., 2008; Weinberg et al., 2008; Uban et al., 2013; Roselli et al., 2020). In a recent human study of youth ages 9 to 16 years, Uban et al. (2017) found that girls with PAE demonstrated lower FA across several white matter tracts, whereas males with PAE showed higher FA (including in the body of the corpus callosum) compared to non-exposed controls, with important group differences by sex in relationships between white matter microstructure and gonadal hormones such as testosterone and progesterone. Similarly, expected positive correlations between age and white matter FA were not observed for males with PAE, suggesting a greater degree of atypicality in white matter microstructure development compared to females. In addition, correlations of DTI metrics with gonadal hormone levels were atypical in PAE, with notable sex differences. Together, data presented here may suggest that white matter microstructure maturation is more atypical in males compared to females with PAE, which may reflect differences in neuroendocrine function, and further research will be important.

Exploratory correlations between change in NDI and ODI and executive function performance may suggest atypical relationships between corpus callosum microstructural maturation and cognitive functioning in youth with PAE. Across regions of the CC, reduction in the bending and fanning of axons (ODI) was associated with better task performance for Control participants but not those with PAE. Similarly, correlation matrices suggested that for Controls, greater negative percent change in NDI (i.e., reductions in the density of white matter fibers) was associated with better task performance. Corpus callosum microstructural integrity has been linked to cognitive performance in children and adolescents with PAE in previous studies, including visual–spatial processing skills (Wozniak et al., 2009), working memory and verbal memory (Gautam et al., 2014), and processing speed and eyeblink conditioning (Fan et al., 2016). Emerging longitudinal research suggests atypical brain maturation-behavior relationships in individuals with PAE as measured by a variety of brain metrics (e.g., cortical thickness, cortical volume, white matter volume, FA/MD) (Moore and Xia, 2021). Our preliminary findings may reflect typical corpus callosum white matter microstructure development in Controls and abnormal experience-dependent development in the PAE group, warranting further exploration in future studies.

Results of exploratory analyses suggest that atypicality in the developmental timing and degree of age-related change in CC microstructure in participants with PAE is associated with dysmorphic facial features. Specifically, dysmorphic features (i.e., philtrum and upper lip) were associated with greater percent change increases in ODI in the posterior CC. Our findings align with several prior studies suggesting a relationship between facial dysmorphology and CC microstructure (Astley et al., 2009; Fryer et al., 2009; Li et al., 2009). However, it is important to note that several other studies found no such relationship (Ma et al., 2005; Wozniak et al., 2006, 2009, 2011). Despite these inconsistencies, a relationship between white matter microstructure and facial dysmorphology is biologically plausible given that midline facial tissues (e.g., eyes, lips, mouth) and midline brain structures (i.e., CC) share the same progenitor cells, which are known to be susceptible to the teratogenic effects of PAE (Sulik, 2005; O’Leary-Moore et al., 2011; Sherbaf et al., 2019). Similarly, prior research has suggested a dose-dependent relationship between the amount of PAE and microstructural abnormalities in the splenium and isthmus of the CC (Fan et al., 2016), and a higher degree of facial dysmorphology has been associated with greater amounts of PAE (Roussotte et al., 2012). As such, our preliminary findings may reflect biologically-meaningful relationships between CC microstructure development and dysmorphic facial features, warranting further study with advanced biophysical modeling of diffusion data (e.g., with NODDI) that may be sensitive to subtle developmental disruptions in white matter microstructure in individuals with PAE. Indeed, preliminary data from our recent study examining long-term outcomes following early choline supplementation in young children with PAE suggested a significant correlation between splenium ODI and the degree of facial dysmorphology (Gimbel et al., 2022), highlighting the need for further investigation.

This study has several important limitations. Due to the modest sample size, subtle (yet potentially meaningful) interaction effects (e.g., sex-by-group, age-by-group) with small to medium effect sizes may have been missed. Given limitations in the sample size, we did not include potential covariates in correlations between change in NODDI metrics and executive function performance. We aim to further explore these relationships in a future study. While the inter-scan interval was significantly longer in the PAE group than in the Control group, the direction of our findings did not change after controlling for the inter-scan interval in analyses. Our sample was also limited in terms of racial and ethnic diversity, resulting in group differences in racial identities for participants in the PAE and Control groups. While FASD is known to affect individuals of all racial and ethnic backgrounds (Olson et al., 2007), group differences in this study may limit the generalizability of our findings. In this study, prenatal exposure to substances other than alcohol was not exclusionary for the PAE group, as this pattern is common. Although brain-based differences resulting from other prenatal exposure to other substances has the potential to add additional noise to the data in studies of PAE, prenatal exposure to alcohol alone has well-documented direct effects on neurodevelopment (Wozniak et al., 2019). Future studies will benefit from further exploration of potential additive or synergistic effects of prenatal polysubstance exposure on white matter longitudinal trajectories. Additionally, in this study we used linear models of microstructure diffusion trajectories to explore white matter microstructure maturation in PAE as we believed linear models to be appropriate given the duration between scans (17 months on average) and the modest sample size. However, white matter development is known to be non-linear, particularly across larger timescales (Lebel et al., 2019), and future studies may benefit from further exploration of potentially atypical non-linear trajectories of white matter change associated with PAE. In addition, the modest sample size examined in this study may have contributed to our finding of trend-level (after FDR correction) diagnostic group differences in ODI symmetrized percent change in the central CC. Future research will benefit from larger sample sizes providing improved statistical power in order to better characterize white matter microstructure maturation in PAE.

## 5. Conclusion

To conclude, data presented here suggest an atypical amount and timing of age-related changes in white matter microstructure (i.e., axon bending and fanning) in the CC in children and adolescents with PAE compared to typically-developing Controls. These findings add to the existing literature on neurodevelopmental trajectories in PAE and suggest that advanced biophysical diffusion modeling (NODDI) may be sensitive to biologically-meaningful microstructural changes in the CC that are disrupted by PAE. Findings of atypical brain maturation-behavior relationships in PAE highlight the need for further study.

## Data availability statement

The raw data supporting the conclusions of this article will be made available by the authors, without undue reservation.

## Ethics statement

The studies involving human participants were reviewed and approved by Institutional Review Board of the University of Minnesota. Written informed consent to participate in this study was provided by the participants’ legal guardian/next of kin.

## Author contributions

BAG participated in the analysis of the study as well as the writing of the manuscript. DJR and BAM participated in the design and conduct of the study including neuroimaging data acquisition, neuroimaging data inspection and analysis, and the writing of the manuscript. AME and MNR participated in the data collection, analysis, and the writing of the manuscript. MEA participated in the analysis and the writing of the manuscript. EW participated in the writing of the manuscript. SNM, KLJ, EPR, and KOL participated in the design and conduct of the study and the writing of the manuscript. JRW participated in the design and execution of the study, analysis, and the writing of the manuscript. All authors read and approved the final manuscript.

## Funding

This study was supported by National Institute on Alcohol Abuse and Alcoholism: U01AA026102, U24AA014815, U01AA014834, and U24AA014811; the Biotechnology Research Center: P41EB015894, the National Institute of Neurological Disorders and Stroke Institutional Center Core Grants to Support Neuroscience Research: P30 NS076408; and the High Performance Connectome Upgrade for Human 3T MR Scanner: 1S10OD017974.

## Conflict of interest

The authors declare that the research was conducted in the absence of any commercial or financial relationships that could be construed as a potential conflict of interest.

## Publisher’s note

All claims expressed in this article are solely those of the authors and do not necessarily represent those of their affiliated organizations, or those of the publisher, the editors and the reviewers. Any product that may be evaluated in this article, or claim that may be made by its manufacturer, is not guaranteed or endorsed by the publisher.

## Footnotes

^1^ www.cifasd.org
